# Impaired malin expression and interaction with partner proteins in Lafora disease

**DOI:** 10.1016/j.jbc.2024.107271

**Published:** 2024-04-07

**Authors:** Alexander V. Skurat, Dyann M. Segvich, Christopher J. Contreras, Yueh-Chiang Hu, Thomas D. Hurley, Anna A. DePaoli-Roach, Peter J. Roach

**Affiliations:** 1 Department of Biochemistry and Molecular Biology, Indiana University School of Medicine, Indianapolis, Indiana, USA; 2 Lafora Epilepsy Cure Initiative, University of Kentucky College of Medicine, Lexington, Kentucky, USA; 3 Division of Developmental Biology, Department of Pediatrics, Cincinnati Children’s Hospital Medical Center, University of Cincinnati College of Medicine, Cincinnati, Ohio, USA

**Keywords:** Lafora disease, malin, laforin, glycogen, glycogen storage disease, phosphatases, glycogen metabolism

## Abstract

Lafora disease (LD) is an autosomal recessive myoclonus epilepsy with onset in the teenage years leading to death within a decade of onset. LD is characterized by the overaccumulation of hyperphosphorylated, poorly branched, insoluble, glycogen-like polymers called Lafora bodies. The disease is caused by mutations in either *EPM2A*, encoding laforin, a dual specificity phosphatase that dephosphorylates glycogen, or *EMP2B*, encoding malin, an E3-ubiquitin ligase. While glycogen is a widely accepted laforin substrate, substrates for malin have been difficult to identify partly due to the lack of malin antibodies able to detect malin *in vivo*. Here we describe a mouse model in which the malin gene is modified at the C-terminus to contain the c-myc tag sequence, making an expression of malin-myc readily detectable. Mass spectrometry analyses of immunoprecipitates using c-myc tag antibodies demonstrate that malin interacts with laforin and several glycogen-metabolizing enzymes. To investigate the role of laforin in these interactions we analyzed two additional mouse models: malin-myc/laforin knockout and malin-myc/LaforinCS, where laforin was either absent or the catalytic Cys was genomically mutated to Ser, respectively. The interaction of malin with partner proteins requires laforin but is not dependent on its catalytic activity or the presence of glycogen. Overall, the results demonstrate that laforin and malin form a complex *in vivo*, which stabilizes malin and enhances interaction with partner proteins to facilitate normal glycogen metabolism. They also provide insights into the development of LD and the rescue of the disease by the catalytically inactive phosphatase.

Lafora disease (LD, OMIM #254780) is a form of epilepsy that follows an autosomal recessive inheritance pattern. It typically manifests during adolescence and advances to ataxia, cognitive deterioration, dementia, and neuronal degeneration, ultimately resulting in a life expectancy of approximately 10 years from disease onset (1, 2, 3, 4, 5, 6, 7). LD is caused by mutations in either of two genes, *EPM2A* or *EPM2B* (also called *NHLRC1*), with approximately 50:50% distribution (7, 8, 9, 10). Almost all of the mutations that have been experimentally tested affect polysaccharide binding, enzyme activity, or interaction with proteins (11). *EPM2A* encodes laforin, a member of the dual specificity phosphatase family (12, 13). Laforin contains a CBM20 carbohydrate-binding module (14, 15, 16) at the N-terminus and a dual-specificity phosphatase domain (17, 18) at the C-terminus. Laforin dephosphorylates complex carbohydrates like amylopectin and glycogen and mouse models lacking laforin exhibit elevated levels of glycogen phosphate (19, 20). *EPM2B* encodes malin, an E3 ubiquitin ligase containing an N-terminal Ring finger domain followed by six NHL domains (21). A hallmark of LD is the accumulation of Lafora bodies (LBs) in muscle, heart, skin, and most notably in astrocytes and neurons (22, 23, 24). LBs are insoluble polyglucosan deposits that contain poorly branched and hyper-phosphorylated glycogen (25, 26, 27, 28, 29, 30) and are considered causative of the disease. Although not initially appreciated, it is now clear that LD is a glycogen storage disease.

Glycogen is a branched polymer of glucose that acts as a reserve of glucosyl units, to be used for anabolism or as a source of energy (31, 32). In mammals, the two major tissue deposits of glycogen are the liver and skeletal muscle, but many organs, notably the brain, also synthesize the polysaccharide. The bulk synthesis of glycogen is catalyzed in the cytosol by glycogen synthase (GYS), in concert with the branching enzyme (GBE) which introduces branches approximately every 13 glucose residues (31, 33). Cytosolic glycogen breakdown is mediated by glycogen phosphorylase (PYGM) and the debranching enzyme (AGL). Glycogen contains trace amounts of covalently attached phosphate (34, 35, 36), with ratios of one phosphate per 500 to 2000 glucoses depending on the tissue source (20, 37). In addition to the cytosolic pathway, glycogen is also degraded within the lysosome through direct hydrolysis to glucose by lysosomal α-glucosidase (acid maltase, GAA) (38, 39, 40). The physiological importance of lysosomal glycogen degradation is underscored by Pompe disease, which is caused by inactivating mutations in GAA and results in a wide spectrum of symptoms with associated lysosomal glycogen accumulation (41, 42). Aberrant glycogen stores are associated with numerous disease states, from type 2 diabetes to classic glycogen storage diseases (GSDs) (25, 43, 44). Although abnormal glycogen can be rationalized in some GSDs, such as Adult Polyglucosan Body disease (45) and Tarui disease (25, 44, 46), how defects in laforin and malin lead to glycogen overaccumulation and altered structure is not completely understood.

Much effort has been directed at elucidating the functions of laforin and malin and how they contribute to the pathology of LD. Deficiency in laforin and malin in mice recapitulate many, but not all of the abnormalities in patients. Laforin (47) or malin (48, 49, 50) KO mice over accumulate glycogen with long branches and have up to a 10-fold increase in glycogen phosphate levels, characteristics of the insoluble LB (20, 28, 37, 51, 52). Glycogen phosphate is also increased in patients with LD (53). The increased glycogen phosphate in the laforin knockout (LKO) mice demonstrated that laforin dephosphorylates glycogen *in vivo*. In addition, laforin and malin KO mice present widespread neuronal degeneration and are more prone to pharmacologically induced seizures (54, 55, 56).

Accumulation of LB in both neurons and astrocytes is considered causative of the disease. Several laboratories have demonstrated that genetically reducing glycogen accumulation can effectively rescue the phenotype in mice afflicted with LD. In double knockout mice lacking laforin or malin and the regulatory subunit of protein phosphatase 1, protein targeting to glycogen (PTG) (55, 57) or GYS1 (54, 56, 58, 59), glycogen levels and LBs are dramatically suppressed and neurological symptoms alleviated. Even monoallelic deletion of GYS1 in the brain abolishes LB formation and restores neurological functions in laforin or malin knockout mice (54, 56). These results support that LB is causative of the disease.

The increased glycogen phosphorylation in LD led to the hypothesis that hyperphosphorylation resulted in disturbances in glycogen structure and solubility leading to LB formation. This hypothesis has been challenged by studies of transgenically overexpressed laforin inactive mutant mice where the catalytic Cys is mutated to Ser (LCS). An initial report by Chan *et al.* (15) showed that in WT mice, which overexpress LCS, glycogen was increased and LB was formed. Subsequently, Gayarre *et al.* (60) reported that overexpression of LCS in LKO mice abolished the formation of LB in the brain by correcting autophagic defects. More recently Nitschke *et al.* (61) revisited the two mouse models and reported that glycogen chain length was normalized in each model while glycogen phosphorylation was still increased. LB formation was abrogated in the LKO mice, but not in malin KO. The reason for the discrepancy with the first report is not clear. Most importantly these studies have the potential problem that the transgenic protein is expressed at 10- to 100-fold over the endogenous levels (60, 62). This raises a concern that overexpression of laforin (WT or CS) impacts glycogen metabolism and structure and may lead to a misinterpretation of the molecular mechanism underlying LB formation. Consistent with this concern, a high level of wild-type laforin overexpression in cultured cells has been reported to cause LB formation (63). The LCS variant has not been found in LD patients and therefore it is not possible to assess whether lack of phosphatase activity is pathogenic. In any event, a main question still remains: how do laforin and malin control glycogen metabolism and structure?

While it is now widely accepted that laforin is a glycogen phosphatase, the function of malin is less understood. Evidence has been published that Malin functions as an E3 ubiquitin ligase; however, the physiological substrates are not clearly established. Studies *in vitro* and using cultured cells showed that malin or a laforin/malin complex are able to interact and ubiquitinate several proteins involved in glycogen metabolism, including glycogen synthase (64), laforin (65), AGL (66), AMP-activated protein kinase (67), PTG (68), and glycogen phosphorylase (69). Sun *et al.* (69) showed that phosphorylase is ubiquitinated *in vitro* using purified proteins. In addition, they demonstrated that malin expression is decreased in patient lung cancer tissue and glycogen is increased. Overexpression of malin in cancer cells resulted in the translocation of glycogen phosphorylase to the nucleus and increased ubiquitination. Some of these studies led to the proposal that laforin binding to glycogen, recruits malin to ubiquitinate and promote proteosomal degradation of the glycogen metabolizing proteins thereby slowing down glycogen synthesis (68). Thus, in this model, malin deficiency would prevent protein degradation resulting in increased levels of glycogen metabolizing enzymes. However, the protein levels of AGL and PTG are unaltered in malin KO mice (28, 48). These studies argue against malin-mediated degradation of these proteins. Moreover, although laforin and glycogen synthase levels were increased in the brains of malin KO mice (54, 57, 58), they were normalized when glycogen was genetically decreased, even though malin was still absent. Thus, the increase in GS and laforin in malin KO mice may be a result of increased stability due to elevated glycogen levels, loss of degradation due to malin deficiency, or a combination of both. Other studies identified additional potential laforin/malin interacting proteins/substrates, including components of the PI3KC3 complex (70), p62/Sequestosome-1 (71), muscle isoforms of pyruvate kinase (72) and PIP3-dependent Rac exchanger (P-Rex) (73). How these potential targets regulate glycogen metabolism is less clear. Recently, the idea has been raised that ubiquitination by malin affects protein localization, binding partners, and/or activity (69, 74). However, the lack of effective antibodies against mouse malin has been a major impediment to studying malin *in vivo*. To this end, we generated mice that express malin with a C-terminal c-myc tag using CRISPR/Cas9 genome editing. Malin-c-myc-tagged mice were crossed with LCS, carrying the catalytic Cys265 to Ser mutation in the genome, and with laforin knockout mice. We find that malin-myc can readily be detected in extracts of skeletal muscle and brain. Mass spectrometry analyses of anti-myc immunoprecipitates from malin-myc mice revealed that malin interacts with laforin and other glycogen-metabolizing enzymes. Moreover, we show that laforin is required for malin to engage with glycogen-metabolizing enzymes. Notably, these interactions do not depend on laforin activity nor are they mediated by glycogen. The myc tag allows us to explore and analyze malin’s function towards understanding its dynamic behavior in cellular interactions and advancing knowledge of its implications in LD.

## Results

### Generation of the malin-myc mouse model

**Figure 1:**
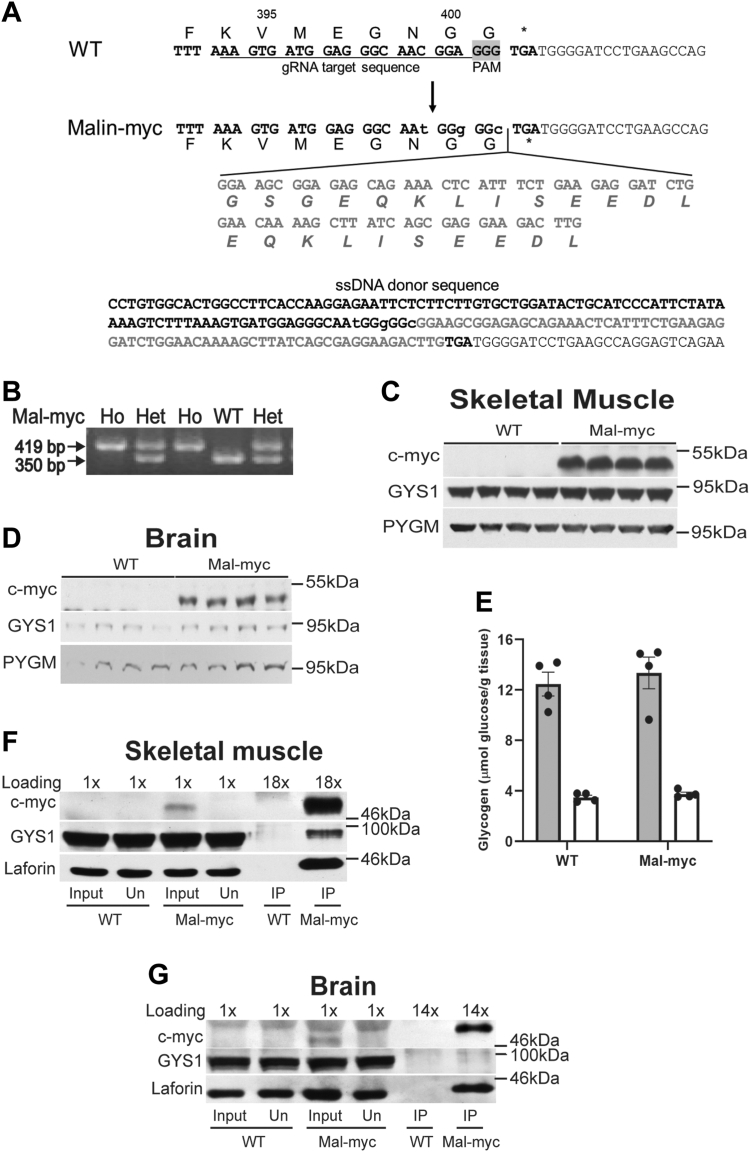
**Design of the malin-myc fusion construct and expression of malin-myc, glycogen synthase, glycogen phosphorylase, and glycogen in skeletal muscle and brain of WT and malin-myc mice and within anti-myc immunoprecipitation from tissues.***A*, CRISPR-Cas9 design strategy to introduce a C-terminal myc epitope tag into the mouse malin gene; gRNA (target sequence underlined), a ssDNA donor sequence containing a linker (GSG), 2× myc-tag and silent mutations (lower case) is indicated; nucleotide and corresponding amino acid sequence are shown, PAM is highlighted in *grey*. *B*, PCR genotyping that identifies WT and malin-myc (Mal-myc) mice produced for this work. WT malin produces a PCR product of 350 bp, whereas the malin-myc fusion gene produces a PCR product of 419 bp. *C* and *D*, immunoblotting of the low-speed supernatant (LSS) of skeletal muscle and brain from 4-month-old mice using antibodies against the c-myc tag, glycogen synthase (GYS1) or muscle isoform of glycogen phosphorylase (PYGM). *E*, glycogen content from skeletal muscle (*black bars*) and brain (*grey bars*) of WT and malin-myc mice. Data from four mice are shown as means ± SEM (n = 4). *F* and *G*, immunoprecipitation of malin-myc from LSS of skeletal muscle and brain of 4-month-old WT and malin-myc mice using anti-c-myc antibodies and Protein-G Agarose. Absorbed proteins were eluted with SDS/loading buffer. Proteins from LSS (Input), unbound fraction (Un), and eluted from Protein-G Agarose (IP) were separated by gel electrophoresis, transferred to nitrocellulose membranes, and blotted with antibodies to c-myc tag, GYS1 or laforin. Loading values (1×, 18× or 14×) indicate the amount of sample loaded relative to the input.

Approximately 50% of LD patients have mutations in the gene encoding malin. However, the role of malin in normal and pathological regulation of glycogen metabolism is not completely understood. Progress has been hindered by the lack of effective antibodies for detecting the malin protein. We sought to express a form of malin that would permit high-sensitivity detection in mouse tissues and that could be immunoprecipitated to identify interacting proteins. Our strategy employed CRISPR-Cas9 technology to introduce to the C-terminus of malin two sequential copies of the c-myc tag for which high-affinity antibodies are available (75) (Fig. 1*A*). PCR genotyping could distinguish between WT, malin-myc heterozygous, and homozygous mice that differed by 69 bp, corresponding to the size of the myc-tag (Fig. 1*B*).

### Expression of malin-myc and immunoprecipitation by anti-myc antibodies

To investigate the expression of malin in the malin-myc mice, we examined skeletal muscle and brain tissues by protein immunoblotting. Unlike the reported Flag-tagged malin that could not be detected in tissue extracts (76), antibodies against c-myc identified a single protein of the predicted molecular weight, ∼46 kDa (including ∼2.7 kDa of the myc tag), in both skeletal muscle and brain extracts from the malin-myc mice with no reactivity present in wild type (WT) extracts (Fig. 1, *C* and *D*). Since there are reports of an interaction between malin and other glycogen-associated proteins (65, 67, 77), we analyzed whether the addition of the C-terminal c-myc tag affected the protein levels of glycogen synthase (GYS1) or glycogen phosphorylase (PYGM). No differences in the levels of GYS1 or PYGM were detected in skeletal muscle or brain when compared to WT mice (Fig. 1, *C* and *D*). Likewise, the addition of the c-myc tag did not affect glycogen accumulation (Fig. 1*E*). We then used the c-myc antibody to immunoprecipitate (IP) malin-myc from low-speed supernatants (LSS) of skeletal muscle and brain homogenates. Protein immunoblotting indicated that the IPs pulled-down malin-myc, while no immunoreactive bands were detected in IPs from the WT animals (Fig. 1, *F* and *G*). Comparison of the c-myc signal in the input and the fraction unbound to the c-myc antibody-agarose demonstrated near complete depletion of the malin-myc protein, indicating that most of the malin had been immunoprecipitated (Fig. 1, *F* and *G*). We then probed for the presence of laforin in the LSS (input) and highly enriched IPs by Western blotting and found that laforin co-immunoprecipitates with malin from skeletal muscle and brain, as did GYS1, although at much lower level in brain (Fig. 1, *F* and *G*). No GYS1 or laforin were detected in IPs from WT supernatants. Interestingly, no apparent depletion of laforin or GYS1 were observed in the unbound fraction suggesting that malin interacts with a small subset of laforin and GYS1 in both tissues (Fig. 1, *F* and *G*).

**Figure 2:**
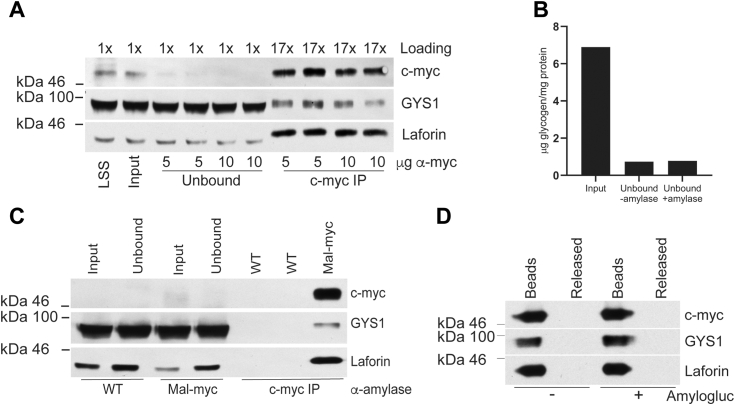
**Near complete immunoprecipitation of malin-myc from skeletal muscle with anti-myc Agarose and effect of α-amylase or amyloglucosidase treatment on immunoprecipitated proteins.***A*, LSS from the skeletal muscle of 4-month-old malin-myc mice was treated with Protein-G Agarose for 1 h followed by centrifugation at 6000*g* for 20 min; the resulting supernatant was designated as Input. Aliquots of this fraction were incubated with anti-myc tag Agarose containing 5 μg or 10 μg of covalently linked anti-c-myc antibody (α-myc) for 18 h at 4 °C. The supernatants obtained after centrifugation and precipitation of anti-myc Agarose were designated as *unbound* fractions. Proteins of LSS, Input, unbound fraction and eluted from anti-myc Agarose with SDS loading buffer (IP) were separated by gel electrophoresis, transferred to nitrocellulose membrane, and blotted with antibodies to c-myc, GYS1 or laforin. Loading indicates the amount of sample loaded relative to the LSS. *C*, the input fraction from WT and malin-myc mice was incubated with or without 0.1 mg/ml of α-amylase followed by immunoprecipitation using anti-myc Agarose. *Input*, *unbound* and eluted proteins were analyzed by Western blot for c-myc, GYS1 and laforin. *B*, glycogen content in the input and unbound fractions from malin-myc samples treated with or without 0.1 mg/ml of α-amylase was measured. *D*, after immunoprecipitation from the skeletal muscle of malin-myc mice, the anti-myc Agarose beads were incubated with (+) or without (−) 0.1 mg/ml of amyloglucosidase for 2.5 h at 37 °C. Supernatants containing released proteins (Released) were collected, beads were washed, and resuspended with SDS loading buffer in the original volume (Beads). Proteins from both fractions were analyzed by Western blot for c-myc, GYS1, and laforin. No difference in the protein levels were observed.

Similar to the results with the unlinked antibody, immunoprecipitation with anti-c-myc-linked to agarose led to an almost complete pull-down of malin-myc, which also enriched laforin and GYS1 from the LSS fraction of skeletal muscle (Fig. 2*A*).

Considering that laforin and GYS1 are known to bind to glycogen, we reasoned that co-IP with malin, could be due to glycogen acting as a bridge. To rule out this possibility, an IP was carried out in the presence of α-amylase, which hydrolyzes alpha-glucosidic linkages in glycogen. Treatment with α-amylase did not prevent the co-IP of malin with laforin and GYS1 (Fig. 2*C*). Interestingly, measurement of glycogen in the unbound fraction, with and without amylase treatment indicated that glycogen had been mostly degraded during the procedure (Fig. 2*B*).

To further assertain that the observed malin-myc interactions were not due to glycogen acting as carrier, the c-myc-agarose beads from skeletal muscle of malin-myc animals were treated with amyloglucosidase an enzyme capable of cleaving terminal glucoses that are α1,4- or α1,6-linked in glycogen. Similar to what we found with α-amylase, digestion of glycogen with amyloglucosidase did not release laforin or GYS1 into the supernatant and three subsequent washes did not reduce the amount of laforin and GYS1 bound to anti-myc agarose (Fig. 2*D*). These results strongly indicate that glycogen is not mediating the co-immunopreciption of malin with laforin or GYS1.

### Identification of malin-myc interacting proteins

**Figure 3:**
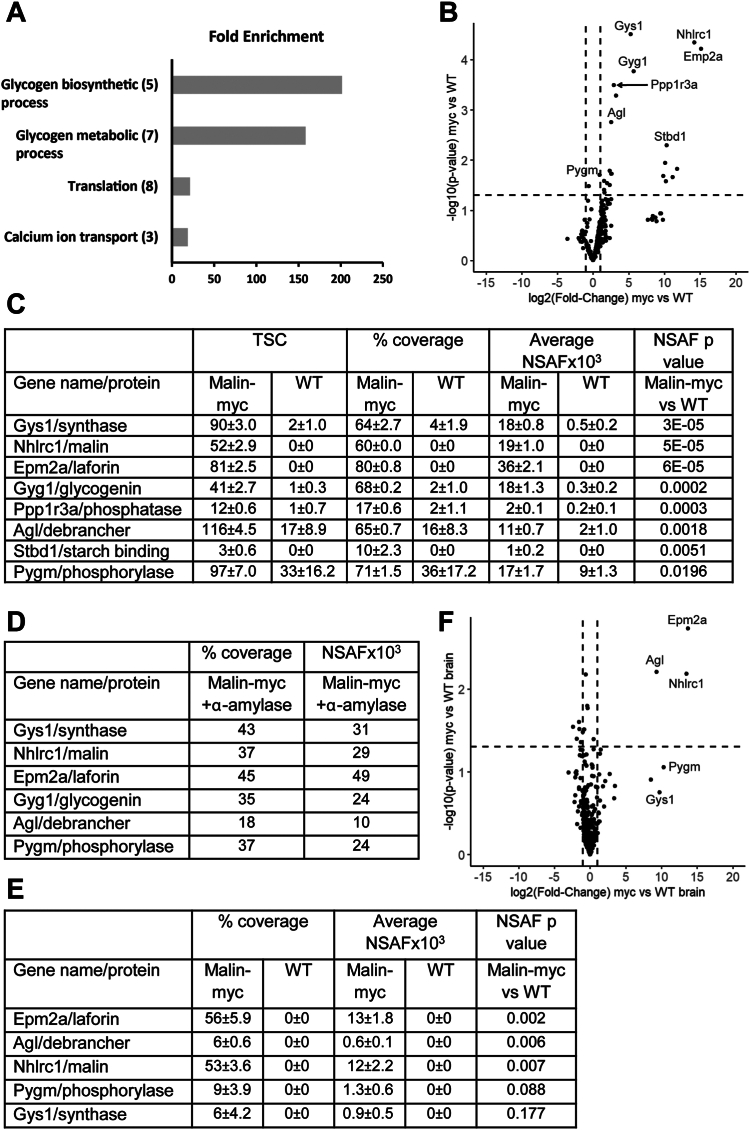
**Quantitative analysis of glycogen-metabolizing proteins identified by mass spectrometry in c-myc IP of skeletal muscle and brain.***A*, DAVID GO term analysis of proteins identified by mass spectrometry in c-myc pull-downs *versus* WT. The 22 proteins for which *t* test *p*-value <0.05 (c-myc *versus* WT) were submitted to the DAVID interface. The histogram displays top Gene Ontology – Biological Process (GO-BP) terms. Bar height represents the Fold Enrichment of the proteins annotated with each term (observed *versus* expected). The numbers in parentheses represent counts of proteins annotated within each group. *B*, volcano plot of skeletal muscle c-myc pull downs *versus* WT displayed as average fold-change (FC) in protein abundance (expressed as log_2_(FC)) on the x-axis *versus* significance (expressed as −log_10_(*p*-value). The thresholds for selecting proteins abundance changes are indicated by the dotted lines. The vertical lines demarcate the log_2_(FC) threshold of ±1 and the horizontal line demarcates −log_10_(*p*-value) equal to 1.3. Significantly enriched proteins (*p* < 0.05) with at least two-fold enrichment in IP from Malin-myc mice (log_2_(FC) >1) are shown in *upper right part* of the graph. The data is shown as *dots* and proteins involved in glycogen metabolism are annotated with their respective gene name. *C*, table of Glycogen-metabolizing proteins identified in IPs from skeletal muscle extracts quantified by total spectrum counts (TSC), protein sequence coverage (% coverage), average NSAF values and *p*-values. *D*, table of glycogen-metabolizing proteins identified in IPs from skeletal muscle extracts treated with α-amylase and quantitated as in panel (*C*). *E*, glycogen-metabolizing proteins identified in IPs from brain extracts quantified as in panel *C*. *F*, volcano plot of proteins identified in IPs from brain tissue of mal-myc mice compared to WT mice, presented as in Panel *B*. Proteins involved in glycogen metabolism are annotated with their respective gene name. Data are average of three independent experiments ± SEM.

To identify malin interacting proteins, c-myc immunopreciptiates from skeletal muscle and brain of the malin-myc mice were subjected to mass-spectrometry analyses. To distinguish malin-interacting proteins from non-specific interactors, IPs from WT mice were used as control. The proteomic analyses identified 267 proteins in the pull-down experiments of skeletal muscle after removal of non-specific interactors (Supplementary File 1). This filtering resulted in 22 proteins that were significantly enriched (*p* < 0.05) in the malin-myc samples *versus* the control samples (Supplementary Enrichment File 2). The 22 proteins with significant enrichment were analyzed using the Database for Annotation, Visualization and Integrated Discovery (DAVID, (78, 79)) web-server. The resulting gene ontology analyses identified “glycogen processes” as the top two functional pathways (Fig. 3*A*). The “glycogen metabolic” cluster contains seven of the most signicantly enriched proteins (NHLRC1, GYS1, EPM2a, Ppp1r3a, PYGM, AGL, Stbd1) in the malin-myc IP (Fig. 3, *B* and *C*; Supplementary Enrichment File 2). It is interesting that glycogenin (GYG1) is not annotated within this particular glycogen metabolic term, rather it is annotated in the GO database as a “glycogen biosynthetic process” protein and appears amongst the five proteins enriched in this GO term cluster (GYG1, GYS1, NHLRC1, EPM2a, and AGL). The third cluster, “Translation”, comprises a translation initiation factor and seven ribosomal proteins which are known to carry-over and to be often detected in LC-MS/MS experiments at low enrichment levels (80, 81). Of the 22 proteins with *p*-value <0.05, there were 21 whose levels were at least 2-fold higher in the malin-myc samples (upper-right quadrant, Fig. 3*B*) and eight of them were the same as those associated with the GO terms “glycogen metabolic process” or “glycogen biosynthetic process” (Supplementary File 2). The LC-MS/MS quantitation of each of the eight “glycogen metabolic/biosynthesis process” proteins identified from the DAVID pathway and by the Volcano plot analyses are presented in Figure 3*C*. Six proteins were detected in high-abundance (NHLRC1, GYS1, EPM2a, GYG1, PYGM, and AGL) and demonstrated good overall sequence coverage (>60% of protein sequence identified), while the other two were detected at lower abundance (Stbd1 and Ppp1r3a) and had lower sequence coverage (Fig. 3*C*). All eight were identified with high confidence in the proteomic analyses. Although AGL and glycogen phosphorylase were found in the IPs from WT muscle, they are still significantly enriched in the malin-myc pull-downs (Fig. 3*C*). Digestion of glycogen with α-amylase during the IP procedures still detected by LC-MS/MS the major proteins identified in the untreated c-myc samples (Fig. 3*D* and Supplementary File 3), confirming that glycogen does not mediate the association between malin and these interacting proteins.

Consistently with the results of the immunoblots, the LC-MS/MS of the IPs from brain tissue were less enriched for some proteins compared to skeletal muscle (Fig. 3*E* and Supplementary File 4). After the removal of non-specific proteins that were present also in the wild-type brain tissue, only three proteins reached statistical significance (*p* < 0.05 and >2-fold change) for enrichment in the malin-myc brain tissues (Fig. 3*E*) and all three are involved in glycogen metabolism (NHLC1, EPM2a and AGL, Fig. 3, *E* and *F*). Although PYGM and GYS1 did not show statistical significance, they were clearly detected in the malin-myc samples and not in the WT (Fig. 3*E* and Supplementary File 4). The identification of fewer significantly enriched proteins is likely due to the lower abundance of glycogen-metabolizing enzymes in the brain. Thus, the results from both the skeletal muscle and brain tissues demonstrate a protein complex between laforin and malin and additional interactors, including AGL, GYS1, PYGM, GYG1, Stdb1, and Ppp1r3a. These malin interactions will help resolve some key questions in the field and have broad implications for malin’s function in health and disease conditions.

### Laforin plays a critical role in malin expression and interacting proteins

**Figure 4:**
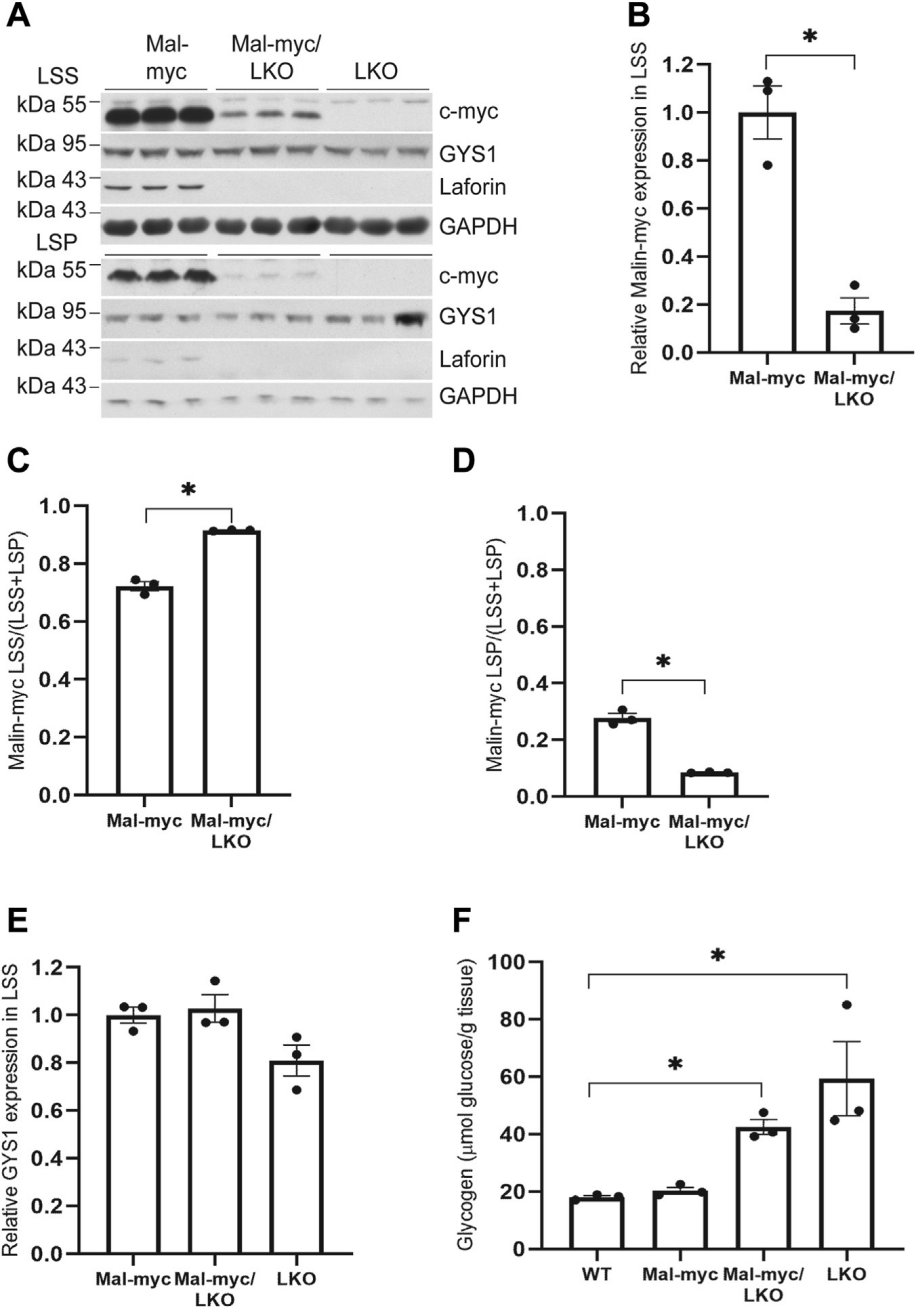
**Levels of malin-myc, glycogen synthase, laforin, and glycogen in skeletal muscle of malin-myc, malin-myc/LKO, and LKO mice.***A*, immunoblot analysis of LSS and low-speed pellet (LSP) from skeletal muscle of 10-month-old mice of the indicated genotype using antibodies to c-myc, GYS1, laforin, or GAPDH as the loading control. Quantitation of malin (*B*–*D*) and GYS1 (*E*) levels in LSS of skeletal muscle from malin-myc and malin-myc/LKO mice. Values are normalized to expression in malin-myc mice. Note the decreased levels of malin in the LKO/malin-myc samples. Malin-myc protein distribution in LSS or LSP, expressed as the ratio of protein in LSS or LSP to the total protein (LSS + LSP) is presented in (*C*) and (*D*), respectively. Data are shown as means ± SEM (n = 3). *Asterisk* (∗) denotes *p* < 0.05 *versus* malin-myc. *F*, total skeletal muscle glycogen in the indicated mouse genotypes, ∗*p* < 0.05 *versus* WT (n = 3).

In order to gain insight into the role of laforin in interactions between malin with its associated proteins, we generated a mouse model expressing malin-myc in the laforin knockout background, malin-myc/LKO. Protein immunoblotting from skeletal muscle of 10-month-old malin-myc mice showed that the majority of malin-myc ∼80% is located in the LSS (Fig. 4, *A*–*D*). This level is decreased by more than 80% in the absence of laforin (Fig. 4, *A* and *B*). Despite the decrease of malin-myc in malin-myc/LKO, the level of GYS1 was not significantly affected (Fig. 4*E*) and both LKO and malin-myc/LKO mice had similar high amounts of glycogen as compared to malin-myc mice (Fig. 4*F*). Thus, the replacement of endogenous malin with malin-myc has no effect on glycogen overaccumulation in LKO mice.

**Figure 5:**
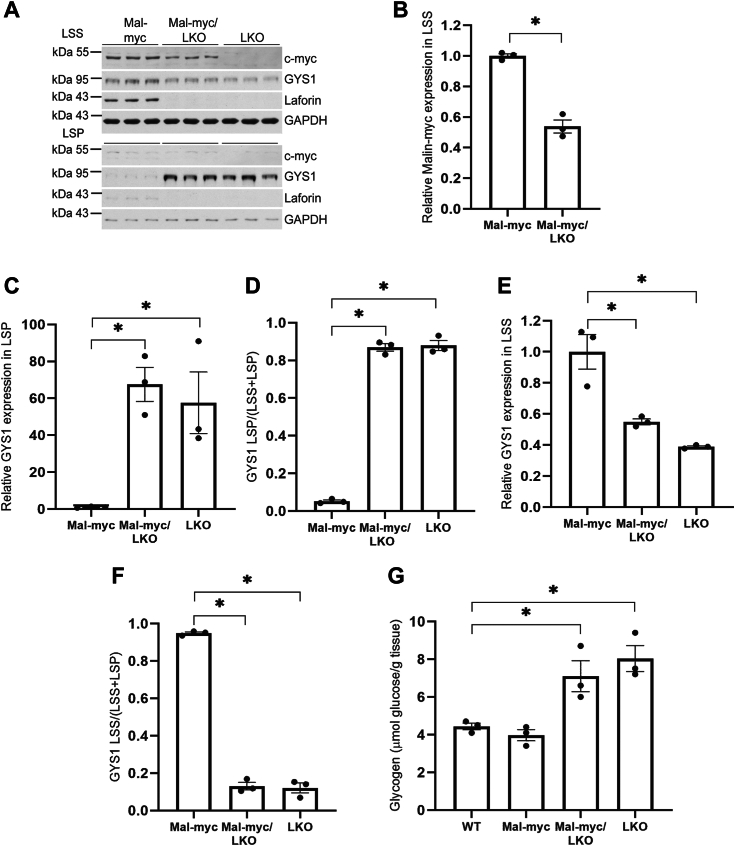
**Expression of malin-myc, glycogen synthase, laforin and glycogen in brain of malin-myc, malin-myc/LKO, and LKO mice.***A*, immunoblot analysis of brain LSS and LSP from 10-month-old mice of the indicated genotype using antibodies to c-myc, GYS1, laforin or GAPDH as loading control. *B*–*F*, quantitation of expression of malin in LSS (*B*) and GYS1 (*C*–*F*) in LSP (*C* and *D*) and LSS (*E* and *F*) of brain from malin-myc and malin-myc/LKO mice. In *B* values are normalized to expression in malin-myc mice. Distribution of GYS1 protein in LSP or LSS is expressed as the ratio of protein in LSP (*D*) or LSS (*F*) to the total protein (LSS + LSP). Note the GYS1 depletion from the LSS (*E* and *F*) and redistribution to the LSP (*C* and *D*) in the malin-myc/LKO and LKO mice. Data are shown as means ± SEM. *Asterisk* (∗) denotes *p* < 0.05 *versus* malin-myc. *G*, total brain glycogen in the indicated mouse genotypes. ∗*p* < 0.05 as compared to WT (n = 3).

In the brain, laforin deficiency also caused a decrease in malin-myc levels but this effect was not as pronounced as in skeletal muscle (Fig. 5, *A* and *B*). As we previously reported (28), GYS1 was highly elevated in the LSP of LKO and malin-myc/LKO brains (Fig. 5, *A*, *C* and *D*). Most of the GYS1 in LKO is found in the LSP, where LBs localize, but the enzyme is not active in this fraction (28). Concomitantly, there is a decrease of GYS1 in the LSS fraction (Fig. 5, *A*, *E* and *F*) (28). Similarly to skeletal muscle, brain glycogen levels in LKO and LKO/malin-myc animals were also significantly higher than in the malin-myc (Fig. 5*G*). Glycogen synthase tracks with glycogen, but the mechanism by which laforin deficiency in the brain but not in the muscle results in increased levels of GYS1 and redistribution to the insoluble fraction is not clear.

**Figure 6:**
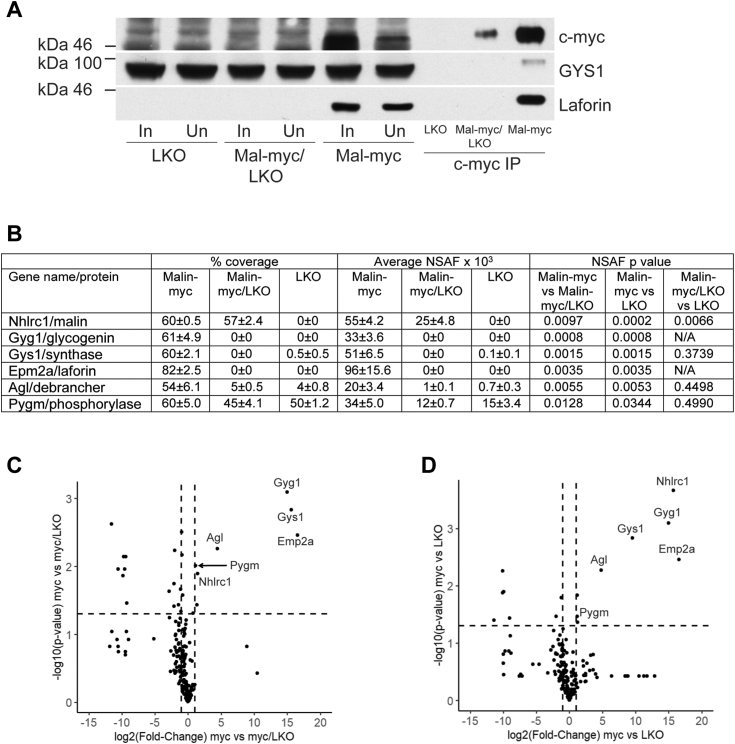
**Analysis of glycogen-metabolizing proteins in IP from skeletal muscle of malin-myc, malin-myc/LKO, and LKO mice.***A*, immunoblot analysis using antibodies to c-myc, GYS1 or laforin of input (In), anti-c-myc unbound fractions (Un), and anti-c-myc IP from 4-month-old mice of the indicated genotype. *B*, table of glycogen-metabolizing proteins identified in IPs by mass spectrometry and presented as percentage protein sequence coverage, average NSAF value and *p*-value. *C* and *D*, volcano plots of proteins identified in IPs from skeletal muscle of malin-myc (myc) compared to malin-myc/LKO and LKO samples displayed by average fold-change (FC) in protein abundance (expressed as log_2_(FC)) on the x-axis *versus* significance (expressed as -log_10_(*p*-value) as in Figure 3*B*. Significantly enriched proteins are shown in the *upper right part* of the graph and those involved in glycogen metabolism are annotated with their respective gene names. The average of three biological replicates ± SEM is shown.

To probe for the effect of laforin deficiency on the interaction of malin with other proteins, immunoblot analysis of c-myc IPs from the skeletal muscle of 4-month-old malin-myc and malin-myc/LKO mice were performed. These analyses showed much lower amounts of malin-myc in IPs from LKO/malin-myc animals, consistent with lower levels of malin-myc in the tissue extracts (Fig. 6*A*). Interestingly, GYS1 was not detected in IPs from malin-myc/LKO despite similar levels in the Input sample. Quantitative mass spectrometry analyses of c-myc immunoprecipitates from c-myc/LKO mice confirmed the lower abundance of malin compared to the control malin-myc samples (Fig. 6*B* and Supplementary File 5), as well as the absence of detectable GYS1 and to near complete absence of most other proteins involved in glycogen processes that were found in the IPs from malin-myc mice, including GYG1, Ppp1r3a and AGL (Fig. 6*B*). Glycogen phosphorylase (PYGM) was detected in the pull-downs from malin-myc/LKO but at lower abundance. The fact that phosphorylase was present also in the pull-down of the LKO mice, where no myc is present, suggests some carry-over or non-specific binding to the agarose due to its high abundance. Volcano plots analyses, still show that the most significantly enriched proteins identified by mass spectrometry in the myc samples compared to the myc/LKO (Fig. 6*C*) or the LKO (Fig. 6*D*) belong to the glycogen metabolism pathway. These results demonstrate that the absence of laforin destablizes the complex formation and that laforin is important to maintain normal malin levels and the interactions with the other partners GYS1, AGL, GYG1, PYGM, and Ppp1r3a.

### Genomically expressed catalytically inactive LaforinC265S is not pathogenic: comparison with the LKO model

**Figure 7:**
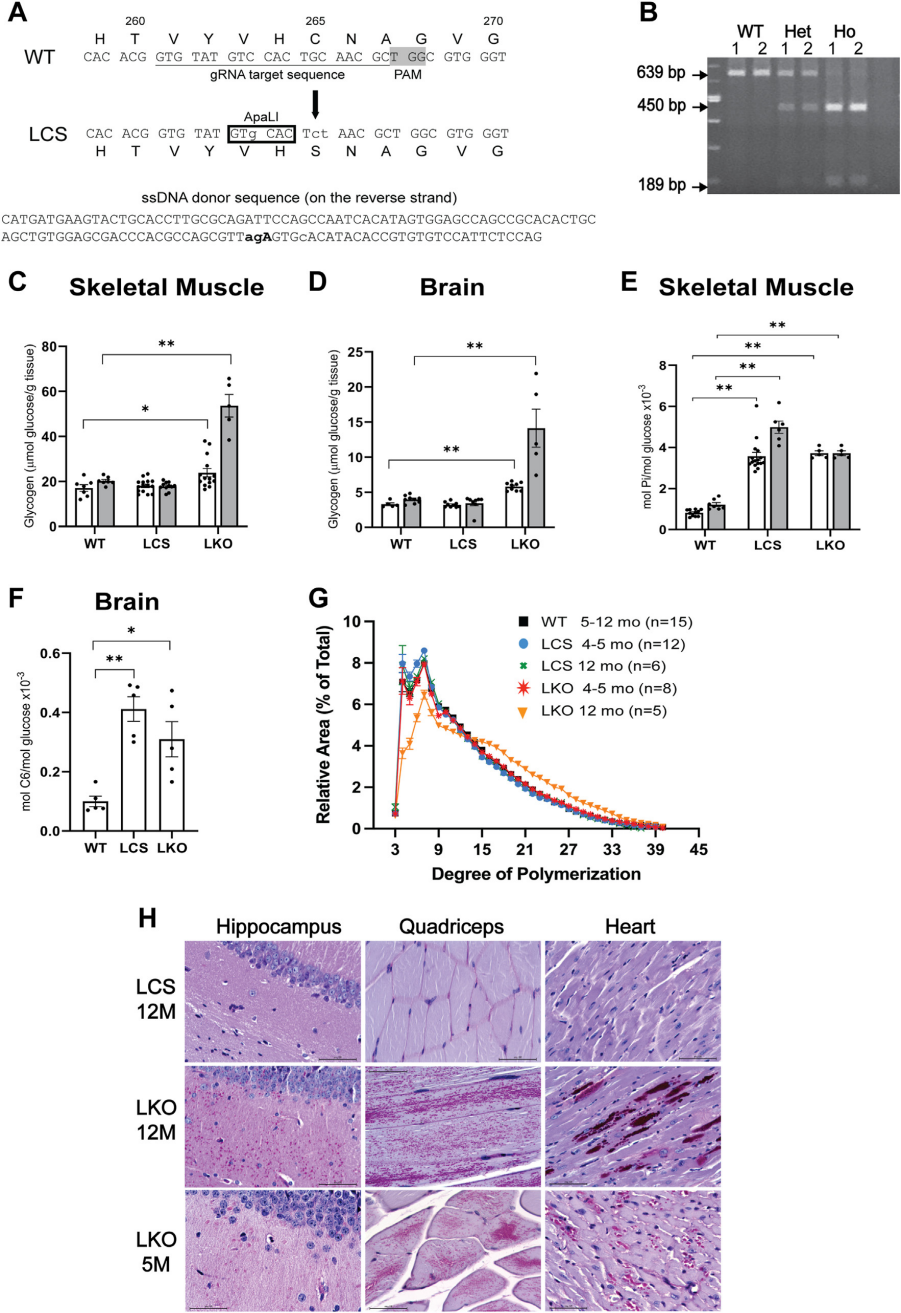
**Design of LaforinC265S (LCS) mutant and comparison of (LCS) with LKO mouse models.***A*, CRISPR-Cas9 design strategy for the introduction of the C265S mutation within the endogenous *EMP2A* gene (Laforin). The gRNA target sequence indicating (a*rrow*) the Cys codon TGC change to the Ser TCT is *underlined* and a ssDNA donor that contains nucleotide changes (*bold lower case*) to introduce C265S and an ApaLI restriction site (*boxed*) is shown. Nucleotides and corresponding amino acid sequences are shown. PAM, highlighted in *gray*. *B*, PCR genotyping of mice WT, heterozygous and homozygous for the LCS mutation. PCR primers were designed to generate a fragment of 639 bp. Digestion with ApaLI results in an uncut fragment of 639 bp for the WT and 450 and 189 bp fragments for the LCS. *C* and *D*, total glycogen content in skeletal muscle (*C*) and brain (*D*) of 5-month-old (*grey bars*) and 12-month-old (*black bars*) WT, LCS and LKO mice, means ± SEM of 7 to 15 mice per genotype. *E*, total glycogen phosphate content in skeletal muscle of 5-month-old (*grey bars*) and 12-month-old (*black bars*) WT, LCS, and LKO mice, means ± SEM of 5 to 11 mice per genotype. *F*, glucose C-6 phosphate in the brain of 12-month-old WT, LCS and LKO mice, means ± SEM of five mice per genotype. *G*, glycogen branch chain length distribution in WT, LKO and LCS mice at 4 to 5 and 12 months of age. On the x-axis the degree of polymerization indicates the number of glucose residues/polymer chain and on the y-axis the percent signal of polysaccharides from 3 to 40 residues. *Symbols* and *colors* denote the different genotypes. Data are shown as means ± SEM of the indicated number of animals. *H*, periodic acid-Schiff (PAS) staining of the hippocampus, quadricep muscle and heart sections of 12-month-old LCS and 5- and 12-month-old LKO mice. *Purple staining* denotes LB. Images were taken at 40× and the bars inside the images denote 50 μm. Representative images from at least four mice are shown. *Asterisks*: ∗*p* < 0.05 *versus* WT; ∗∗*p* < 0.001 *versus* WT.

Prior work with the catalytically inactive form of laforin (C265S, LCS) demonstrated normalization of glycogen levels and reduction of LB formation in LKO mice (15, 60, 61), but these studies were performed using transgenically overexpressed LCS. One caveat of that work was that the transgenic expression of the LCS protein was up to 100-fold over the endogenous laforin levels, which could have led to effects not directly associated with the loss of catalytic activity. Consequently, it was important to characterize a mouse model that expresses laforin C265S from its endogenous promoter to ensure that levels are as close to WT as possible. Utilizing CRISPR-Cas9 technology (Fig. 7*A*) we generated C265S mutants (Fig. 7*B*) to evaluate effects on glycogen metabolism, LB formation, and interaction with malin in the context of physiological gene expression.

Determination of glycogen content in LCS at 4 to 5 and 12 months of age did not show differences as compared to WT mice, wheres LKO mice exhibited a small but significant increase at the younger age and 3- to 4-fold increase at 12 months both in skeletal muscle (Fig. 7*C*) and in the brain (Fig. 7*D*). Total glycogen phosphate in skeletal muscle (Fig. 7*E*) and C-6 phosphate in the brain (Fig. 7*F*) were increased by ∼4-fold at the two ages in both the LCS and the LKO mice, consistent with the lack of catalytic activity. A distinctive feature of LD is the presence of elongated branches in glycogen. Determination of chain length distribution revealed that LCS glycogen was normally branched at both ages analyzed (Fig. 7*G*), whereas branches in the LKO glycogen were normal in the younger mice, but longer at 12 months (Fig. 7*G*) where there was a clear decrease in the proportion of shorter branches from 3 to 15 glucose residues and an increase in longer branches of 15 to 37 residues. Periodic acid-Schiff staining of skeletal muscle, brain, and heart of 12-month-old LCS and 4 to 5 and 12 months LKO mice (Fig. 7*H*) showed no LB in the LCS mice which were clearly abundant in the LKO mice at both ages, even though chain length was normal at 4 to 5 months. This result raises questions about the strong claim (61) that glycogen chain length, not phosphorylation, is the driver for LB formation and LD. Against this argument is also the phenotype of the APBD, where chain length is elongated, but neither the patients nor the mouse models develop LB or LD. The implication of whether glycogen length or phosphorylation is the driver for LB formation and LD is presented in the discussion.

### Laforin catalytically inactive phosphatase interacts with malin and partner proteins

**Figure 8:**
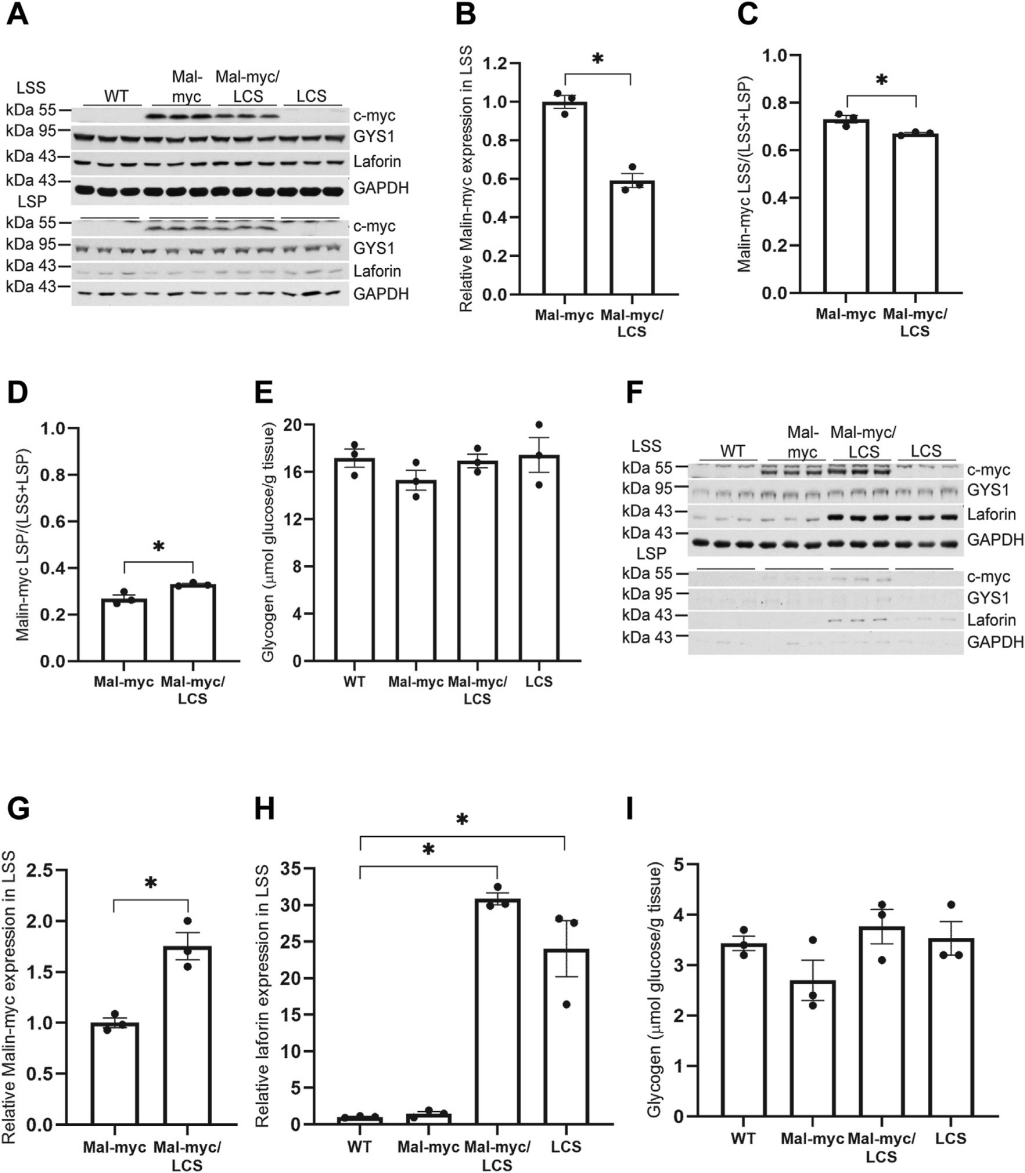
**Expression of malin-myc, glycogen synthase, laforin and glycogen levels in skeletal muscle and brain of WT, malin-myc, malin-myc/LCS, and LCS mice.***A*, immunoblot analysis of LSS and LSP from skeletal muscle of 10-month-old mice with indicated genotype using antibodies to c-myc, GYS1, laforin, or GAPDH as loading control. *B*, quantitation of malin expression in LSS of malin-myc and malin-myc/LCS mice. Values are normalized to expression in malin-myc mice. *C* and *D*, quantitation of malin-myc distribution in LSS (*C*) or LSP (*D*), expressed as the ratio of protein in LSS or LSP to the total protein (LSS + LSP). *E*, total skeletal muscle glycogen in 10-month-old mice of the indicated genotypes. *F*, immunoblot analysis of LSS and LSP from brain of 10-month-old mice with indicated genotype using antibodies to c-myc, GYS1, laforin or GAPDH as loading control. *G* and *H*, quantitation of malin expression in LSS of brain from malin-myc and Malin-myc/LCS mice (*G*) and laforin/LCS expression in LSS of brain from mice of indicated genotype (*H*). Note the high level of LCS expression associated with the brain soluble fraction (*F* and *H*). *I*, total brain glycogen in 10-month-old mice of the indicated genotypes. Representatives of three immunoblots for skeletal muscle or brain are shown and data are presented as means ± SEM (n = 3). *Asterisk* (∗) denotes *p* < 0.05 as compared to malin-myc.

We next sought to investigate whether laforin’s catalytic activity plays a role in malin expression and its interactions with other proteins by crossing the malin-myc mouse with the LCS model to generate malin-myc/LCS doubly homozygous mice. In the skeletal muscle of 10-month-old mice, malin-myc/LCS led to a 40% reduction of malin-myc protein (Fig. 8, *A*–*D*) but no change in laforin (LCS), GYS1 or glycogen (Fig. 8, *A* and *E*) as compared to LCS or WT. The majority of the laforin protein was in the LSS, as were malin-myc and GYS1 (Fig. 8, *A*–*C*), consistent with no LB formation.

In contrast to the skeletal muscle, in the brain, the catalytically inactive mutant elicited an approximate 80% increase in the malin protein in the double mutant mice as compared to the malin-myc mice, and it was mostly confined in the souble fraction (Fig. 8, *F* and *G*). Although in skeletal muscle, the level of laforin was similar to WT, in the brain, it was increased close to 20-fold and it was mostly present in the LSS (Fig. 8, *F* and *H*). The slight increase of laforin in the LSP of malin-myc/LCS compared to LCS (Fig. 8, *F* and *H*) may be due to trapping in the pellet of the somewhat higher levels in the LSS. Despite the increased levels of LCS, GYS1, and glycogen levels in the brain were unaltered (Fig. 8, *F* and *I*) indicating that a high level of LCS mutant most likely does not affect glycogen metabolism.

**Figure 9:**
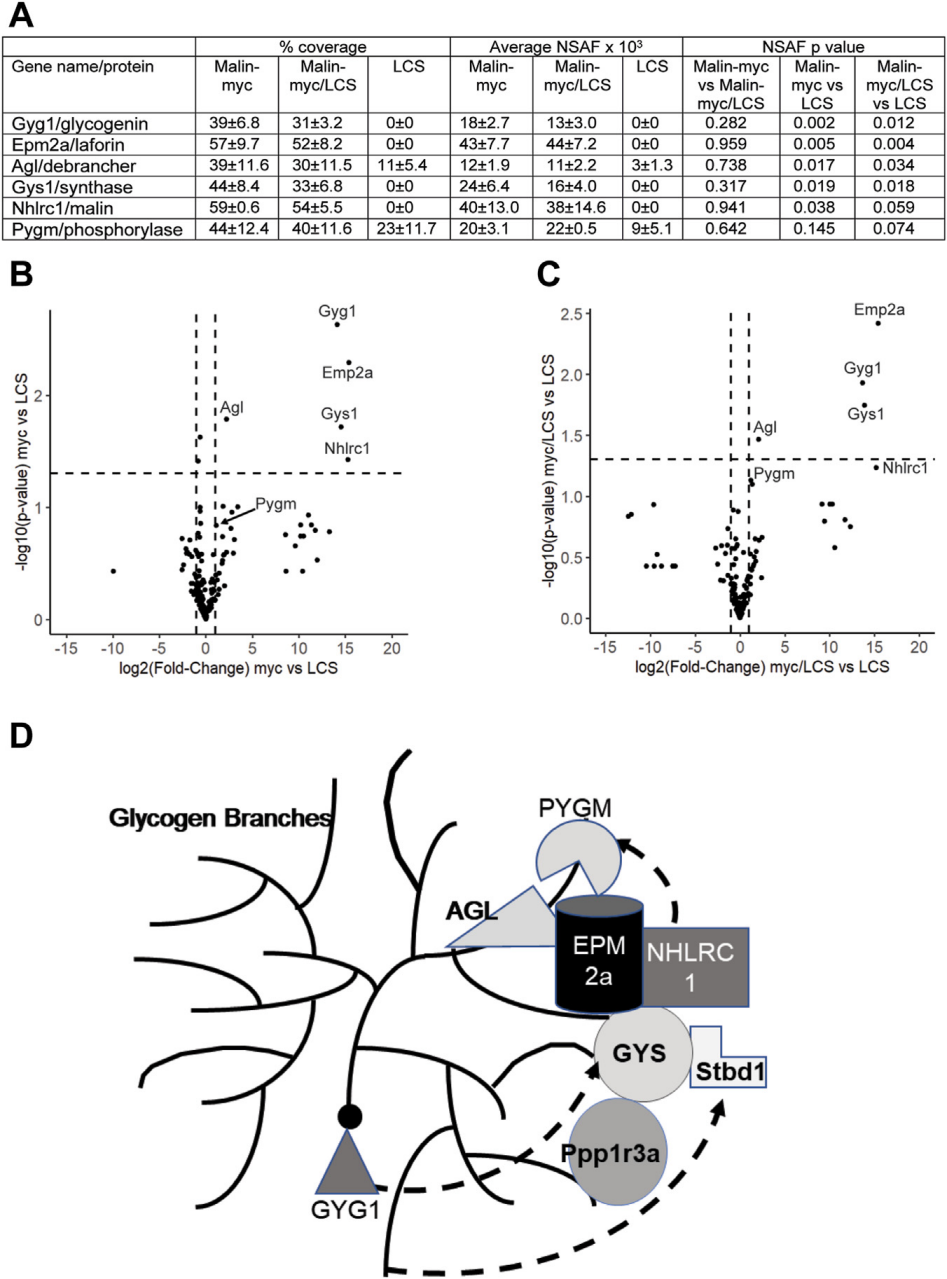
**Analysis of malin-interacting proteins in IP from skeletal muscle of malin-myc, malin-myc/LCS and LCS mice and model of interacting proteins.***A*, table of glycogen-metabolizing proteins identified in IPs by mass spectrometry and presented as percent protein sequence coverage, average NSAF value, and *p*-value. *B*, volcano plot displaying the average fold-change (FC) in protein abundance (expressed as log_2_(FC)) on the x-axis *versus* significance (expressed as −log_10_(*p*-value) in skeletal muscle of malin-myc (myc) compared to LCS mice. *C*, volcano plot for proteins identified in IPs from skeletal muscle of myc/LCS mice compared to LCS mice. The data is shown as *dots* and proteins involved in glycogen metabolism are annotated with their respective gene name. The average of three biological replicates ± SEM are shown. *D*, a model depicting malin-interacting proteins in the context of glycogen metabolism. Proteins identified in this study EPM2a, NHLRC1, AGL, GYS1, PYGM, GYG1, Stdb1, and Ppp1r3a are shown as interacting with each other in a cluster, some can also associate directly with glycogen. Glycogen branches are shown with *solid lines*. *Dotted lines* indicate known or proposed interactions.

To investigate whether interaction with partner proteins was affected by the catalycally inactive laforin, anti-c-myc immunoprecipitates were performed from skeletal muscle of 4-month-old malin-myc, malin-myc/LCS and LCS mice and subjected to LC-MS/MS analyses. Mass spectrometry analyses of the immunopreciptates identified the same proteins in skeletal muscle of malin-myc and malin-myc/LCS animals and similar abundance of each of the malin-interacting proteins (Fig. 9*A* and Supplementary File 6). A comparison of the proteomic results from the malin-myc and malin-myc/LCS demonstrates no significant differences between the two sample sets. Consistently, Volcano plots of the malin-myc or malin-myc/LCS *versus* LCS samples show enrichment of the most prominent glycogen metabolizing enzymes (GYG1, EPM2a, AGL, GYS1 and NHLC1; Fig. 9, *B* and *C*) as observed in the malin-myc samples relative to WT (Fig. 3, *B* and *C*). Therefore, we conclude that the catalytically inactive laforin may decrease the levels of malin but it does not abrogate the interaction of malin with partner proteins. Thus, catalitically inactive laforin still forms a complex with malin which is necessary for the association of the partner proteins.

## Discussion

Malin is one of the two proteins whose gene is mutated in LD, a devastating juvenile neurodegenerative condition that results in death within 10 years of diagnosis.

Although the function of laforin as a glycogen phosphatase is widely accepted, the function of malin is not fully understood. Studies of malin have been hampered by the lack of antibodies able to detect the protein. Several laboratories, including our own, have failed to generate malin antibodies suitable for biological measurement. To overcome this problem, we introduced a myc-tag to the C-terminus of the endogenous mouse malin gene. The presence of the tag allowed robust detection of malin in different tissues and subcellular fractions. Analyses of glycogen metabolizing enzymes showed that the presence of the c-myc tag did not affect their expression nor glycogen levels in skeletal muscle or brain (Fig. 1).

Immunoprecipitation (IP) followed my mass spectrometry of endogenously expressed proteins is one of the most biologically relevant techniques to identify protein-protein interactions. Our study demonstrates that malin is associated with several proteins known to be involved in glycogen metabolism: EPM2a, GYS1, GYG1, AGL, PYGM, Stbd1, and Ppp1r3a. For Ppp1r3a and Stbd1 only a small number of peptides were detected (Fig. 3*C* and Supplementary File 1). This can be explained by the low level of expression of these proteins in skeletal muscle and/or by weak interactions with malin. Four of these proteins, GYS1, GYG1, AGL, and PYGM were also found in proteomics studies of glycogen isolated from 3T3L1 adipocytes (82) and from mouse and rat liver (83), where liver-specific isoforms of glycogen synthase (GYS2) and glycogen phosphorylase (PYGL) were detected. It is particularly noteworthy that malin was not identified in those studies and laforin was only present in mouse glycogen at very low abundance and could not be eluted from glycogen by malto-oligosaccharide treatment (82, 83).

Since malin forms a complex with laforin and laforin contains a carbohydrate-binding domain, we hypothesized that interaction of malin/laforin with other glycogen-bound proteins could be mediated by glycogen itself. However, we found that α-amylase treatment of the LSS from skeletal muscle did not disrupt completely the interaction of malin with laforin, GYS1, GYG1, and AGL. Additionally, treatment of malin-myc IPs with amyloglucosidase did not release the pulled-down proteins from anti-myc agarose-beads. These results indicate that malin or the malin/laforin complex pulled down GYS1, GYG1, and AGL without glycogen acting as a bridge molecule, although we cannot categorically exclude the presence of some residual glycogen. However, *in vivo* the majority of these proteins either in complex or individually associated with glycogen are involved in glycogen metabolism. One possibility is that GYS1 and GYG1 found in the IP is the result of strong binding of GYG1 with GYS1 as recently shown in CryoEM structures (84, 85, 86, 87) and that malin directly interacts with only one member of this pair. Our mass spectrometry analyses did not identify the glycogen branching enzyme (GBE) or PTG, both of which were proposed to be substrates of malin from *in vitro* studies (68, 69).

One intriguing finding of this study is the reduced expression of malin in skeletal muscle and brain of LKO mice (Figs. 4 and 5). Moreover, this effect was accompanied by a complete loss of malin-interacting proteins in the LC-MS/MS of the malin IPs (Fig. 6, *A* and *B* and Supplementary File 5). Therefore, we propose that laforin plays multiple roles in malin function; (1) it interacts with malin and enhances its stability. Indeed, numerous instances exist where proteins within a complex exhibit decreased stability in the absence of their binding partner. (2) it participates in the interactions between malin and glycogen metabolizing partner proteins and, (3) its carbohydrate-binding domain associates with glycogen and recruits malin and interacting partners to the glycogen particle (Fig. 9*D*). These findings help to clarify our understanding of why mutations in either laforin or malin, two functionally different proteins, give rise to abnormal glycogen structure and a similar disease phenotype in LD. The result of mutations in either gene results is either a non-functional malin such as in the malin KO and/or significantly decreased malin levels such as in the LKO.

It is interesting that only a small subset of laforin and GYS1 interact with malin (Figs. 1, *F* and *G* and 2, *A* and *B*) indicating that GYS1 and laforin are much more abundant, whereas all of the malin in the cell appears to be in a complex with laforin or with laforin and GYS1, possibly forming a ternary complex. Indeed, in laforin KO mice, malin does not Co-IP with GYS1 suggesting that laforin bridges an interaction between malin and GYS1 (Fig. 6, *A* and *B*). Although the decrease of malin protein levels in LKO mice may be explained by the lack of malin-laforin complex formation, which decreases malin’s stability, even an 80% decrease is not sufficient to affect the high levels of non-interacting GYS1 and therefore glycogen accumulation. It is unclear though what the function of malin-free laforin is and how it contributes to glycogen metabolism. The role of these complexes between malin, laforin, GYS1 and other partner proteins and the apparent discrepancy of malin-laforin stoichiometry warrants further investigation.

This study also aimed to address the role of the catalytic activity of laforin in glycogen metabolism using our first model of genomically expressed inactive laforin (LCS). Genomic LCS did not alter glycogen levels or chain length but it did cause glycogen hyperphosphorylation with no LB body formation (Fig. 7), similar to what was reported with the transgenically overexpressed protein (60, 61). This led Nitschke *et al.* (61) to conclude that chain length but not phosphorylation is critical for LB formation. However, a number of arguments can be raised against this claim: (1) LB formation requires increasing glycogen accumulation, which does not occur in the LCS mice; (2) LKO mice of young age, 4 to 5 months, show some increase in glycogen accumulation, have increased glycogen phosphate but normal chain length, yet still form LBs (Fig. 7); (3) Perhaps most importantly, patients and mouse models of APBD, where the defect is in the branching enzyme, have increased glycogen and chain length, but they do not form LBs and do not develop LD. Altogether, these data strongly support that LB formation and development of LD require that all three parameters, glycogen levels, phosphorylation, and chain length be abnormal. Therefore, increased chain length, by itself, is not sufficient to form LB and LD.

Catalytically inactive laforin still forms a complex with malin and interacts with other glycogen-metabolizing enzymes (Fig. 9 and Supplementary File 6). Although in skeletal muscle extracts, the malin levels seem to be somewhat decreased while laforin levels are unaffected, (Fig. 8, *A* and *B*), mass spectrometry of the IPs did not demonstrate differences in the abundance of the glycogen-associated proteins identified (Fig. 9*A*). These results suggest that C265S substitution in laforin may weaken the interaction between laforin and malin, leading to partial destabilization and degradation of malin in skeletal muscle. However, in brain, the LCS mutant was dramatically overexpressed which caused a ∼60% increase in the levels of malin (Fig. 8, *F* and *G*). The reason why LCS laforin is elevated in the brain, and not in skeletal muscle, is unclear.

Recently, malin-interacting proteins were identified *in vitro* by incubation of recombinant tagged malin with HEK293 cell lysates, separating the pulled-down proteins on SDS-PAGE, excising some putative bands, and analyzing them by mass spectrometry (69). Several proteins were identified, including laforin, AGL, glycogen branching enzyme, brain isoform of glycogen phosphorylase, heat shock proteins HSP70 and HSP90, and elongation factor 2. In this study, we confirm that laforin, AGL and glycogen phosphorylase are malin-interacting proteins but we did not identify glycogen branching enzyme. As for HSP70 and HSP90, we consistently observed some members of these protein families in the LC-MS/MS analyses, but they were present equally in both malin-myc and WT samples. Similarly, elongation factor 2 was not specifically enriched in malin-myc IPs (Supplementary File 1).

While this article was in preparation, a study describing a mouse model in which the native malin gene is tagged with the FLAG sequence was published (76). The authors could not detect Flag-malin in tissue extracts or in high-speed pellets, where most of the glycogen is present. Enrichment by IP was required for the detection of malin. One of the findings in this study was the association of malin with glycogen only in the presence of laforin, consistent with our observation of decreased malin protein in the LKO. Our data indicate that malin in complex with laforin binds to specific proteins associated with glycogen although direct binding to glycogen cannot be definitively excluded even though glycogen degradation did not abolish the interacting proteins pulled down.

The inability of partner proteins to interact with malin in the absence of laforin, illuminates the key role that the laforin/malin complex plays in glycogen metabolism As shown in the model (Fig. 9*D*), we propose that binding of laforin to glycogen through its glycogen-binding domain recruits malin to form a complex that then interacts with a number of glycogen metabolizing proteins (31): GYS1, the glycogen elongating enzyme interacts directly with the GYG1, the glycogen initiating enzyme (86, 87) and with Ppp1r3a, the regulatory subunit of a phosphatase that dephosphorylates GYS1; Stbd1 can interact with GYS1 and laforin and is involved in glycogen degradation (88); AGL1 removes branches in glycogen, and PYGM degrades glycogen from the non-reducing ends. Although some of these proteins can bind directly to glycogen and may not be completely and always associate with the cluster, we propose that this multiprotein complex is essential for normal glycogen metabolism and the absence of either malin or laforin prevents the association of these critical enzymes resulting in abnormal glycogen accumulation.

In summary, our results confirm an important role for the laforin-malin complex in regulating glycogen metabolism and have established two new mouse models, malin-myc and LCS, that will aid the research community in elucidating the molecular mechanisms of LB formation in LD.

## Experimental procedures

### Chemicals and reagents

Amyloglucosidase (*Aspergillus niger*, Cat No A7420) and α-amylase (*Bacillus* species, Cat No A6380) were from Sigma-Aldrich. Rabbit c-myc antibody immobilized to agarose (Cat No RMYC-145D) and rabbit c-myc tag antibody (Cat No RMYC-45A-Z) were from Immunology Consultants Laboratory. Monoclonal myc Antibody (9E10):sc-40 was from Santa Cruz. Anti-laforin antibody was from Abnova (Cat No H00007957-M02), anti-GYS antibody was from Cell Signaling Technology (Cat No 3886) and the anti-glyceraldehyde-3-phosphate dehydrogenase antibody was from Biodesign International (Cat No H86504M). The antibodies to glycogen phosphorylase (PYGM) were a gift from Dr Gerald Carlson, University of Kansas Medical Center (89, 90). Trypsin/LysC Gold mass Spectrometry grade was from Promega Corporation (Cat No V5071).

### Mouse lines

The mouse models used in this study are laforin knockout (LKO, (47)), and malin-myc and Laforin C265S (LCS) generated in this study at the Transgenic Animal and Genome Editing Core, Cincinnati Children’s Hospital Medical Center. All animal studies were conducted in accordance with federal guidelines and were approved by the Institutional Animal Care and Use Committees of the Cincinnati Children’s Hospital Medical Center and Indiana University, School of Medicine. The mice were housed in a 12:12 h light-dark cycle and were given *ad libitum* access to food and water. Male and female mice were used interchangeably.

### Malin-myc mice

The malin-myc mice were engineered by CRISPR technology to insert an in frame double c-myc tag at the C-terminus, preceded by a short GSG linker (donor sequence below Fig. 1*A*). The single-guide RNA (sgRNA Eg14 target sequence; AAGTGATGGAGGGCAACGGA, Fig. 1*A*) was selected according to the location proximal to the target site and to the on- and off-target scores from CRISPOR.org (91) and transcribed *in vitro* from a PCR-generated DNA template containing a T7 promoter and the sgRNA scaffold, using the MEGAshortscript T7 kit (ThermoFisher), purified by the MEGAclear Kit (ThermoFisher), and stored at −80 °C. The injection mix was prepared by incubating the sgRNA and Cas9 protein (IDT) at 37 °C for 10 min to form ribonucleoproteins followed by addition of the 198 nt single-stranded DNA donor oligo (IDT Ultramer) CCTGTGGCACTGGCCTTCACCAAGGAGAATTCTCTTCTTGTGCTGGATACTGCATCCCATTCTATAAAAGTCTTTAAAGTGATGGAGGGCAAtGGgGGc***GGAAGCGGAGAGCAGAAACTCATTTCTGAAGAGGATCTGGAACAAAAGCTTATCAGCGAGGAAGACTTG***TGATGGGGATCCTGAAGCCAGGAGTCAGAA. Bold italics denote the GSG linker and the 2× myc tag sequences; lower cases denote the silent mutations introduced to prevent sgRNA targeting and re-cutting (Fig. 1*A*). The final concentration was 50 ng/μl sgRNA, 100 ng/μl Cas9 protein, and 100 ng/μl donor oligo. The mutant mice were generated by injection of the mix into the cytoplasm of 60 one-cell-stage embryos of C57BL/6 genetic background by a piezo-driven microinjection technique (92). Injected embryos were immediately transferred into the oviductal ampulla of two pseudopregnant CD-1 females. The 13 pups born were genotyped by PCR (forward primer CTCTGCTGTGACCTTCGATCAC and reverse primer ACTGCCTTAGTAACTGCTTTTGCC) and Sanger sequencing, and three mice were found to carry the correct insertion. These mice were crossed with C57BL/6 and the progeny was genotyped by PCR of tissue biopsies (Fig. 1*B*). Intercrossing of male and female heterozygous for malin-myc generated WT (wild type), malin-myc heterozygous and homozygous mice as determined by PCR (Fig. 1*B*). For further confirmation, the PCR amplicons WT 350 bp and Malin-myc 419 bp, differing by the size of the tag, were gel purified and subjected to Sanger sequencing. Experimental animals were produced from those carrying the confirmed insertion and genotyped by PCR. All mice used in this study are homozygous for the c-myc insertion.

### LaforinC265S mice

To generate the LaforinC265S (LCS) knockin mice, where the laforin catalytic Cys is mutated to Ser, three sgRNAs (sgRNA471, 472, and 473) that target around the intended mutation site were selected, according to the on- and off-target scores from CRISPOR.org (91). sgRNA vector construction was described previously (93). Briefly, pairs of complementary DNA oligos with compatible overhangs were annealed and cloned into a pX458 vector that carries a U6 promoter to drive sgRNA expression and a ubiquitously expressed promoter to drive Cas9-2A-GFP expression (Addgene plasmid #43138). sgRNA editing activity was evaluated in mouse mK4 cells (94) by the T7E1 assay (New England Biolabs), and compared to the control *Tet2* sgRNA that has been shown to edit the mouse genome efficiently (95). SgRNA472 (GTGTATGTCCACTGCAACGC) Figure 7*A* had the highest cutting activity (not shown) and was *in vitro* transcribed from a PCR-generated DNA template containing a T7 promoter and the sgRNA scaffold, using the MEGAshortscript T7 kit (ThermoFisher), purified by the MEGAclear Kit (ThermoFisher), and stored at −80 °C. The injection mix, was prepared by incubating the sgRNA and Cas9 protein (ThermoFisher) at 37 °C for 10 min to form ribonucleoproteins and then adding the 126 nt single-stranded DNA donor oligo (IDT Ultramer) CATGATGAAGTACTGCACCTTGCGCAGATTCCAGCCAATCACATAGTGGAGCCAGCCGCACACTGCAGCTGTGGAGCGACCCACGCCAGCGTT***AGA***GTGcACATACACCGTGTGTCCATTCTCCAG, the sequence in the reverse orientation Figure 7*A*. Bold italics denote the Cys codon TGC was replaced with the Ser codon TCT; the lower case denotes a silent mutation that was introduced to generate an ApaL1 restriction site to facilitate PCR genotyping which also increased the number of mismatches to block sgRNA re-cutting (Fig. 7*A*). The final concentration of the mix was 100 ng/μl sgRNA, 200 ng/μl Cas9 protein, and 100 ng/μl donor oligo. The mutant mice were generating following the procedure described above for the malin-myc mice except that 80 one-cell-stage embryos were injected and transferred into three pseudopregnant CD-1 females. The 21 pups born were genotyped by PCR, enzyme digestion, and Sanger sequencing. Three of the mice appeared to be homozygotes for the mutation. Two were intercrossed and one was crossed with C57BL/6. PCR genotyping and sequencing of the PCR amplicons confirmed the mutation. The PCR primers: forward AGCCACTGCCAGATCTTGGAAAACC and reverse GGAAGAGTGAACCTTCCCGAAC generate a product of 639 bp for both the WT and the LCS. Digestion with ApaL1 produces fragments of 450 bp and 189 bp for the mutant, allowing clear identification of the genotype (Fig. 7*B*). All LCS mice used in this study are homozygous for the mutation.

### Malin-myc/LKO and malin-myc/LCS

To generate the double mutants, Malin-myc/LKO or malin-myc/LCS mice, malin-myc heterozygous mice were crossed with either LKO or LCS homozygous. Male and female double heterozygotes were then intercrossed to produce for each pair WT, individual single homozygous and double homozygous mice that were used for the study. All mice were PCR genotyped using the procedure described above for the malin-myc and LCS and established procedures for the LKO (47).

### Mouse tissue harvesting

WT, malin-myc, malin-myc/LKO, LKO, malin-myc/LCS and LCS mice 4 months or 10 months of age, were euthanized by cervical dislocation, the heads were decapitated directly into liquid nitrogen, and skeletal muscle was rapidly excised, immersed in liquid nitrogen, and stored at −80 °C until use. For brain tissue harvesting, the skulls were cracked open under liquid nitrogen, the brain was removed and powdered under liquid nitrogen. This was necessary to avoid rapid post-mortem glycogen degradation.

### Preparation of mouse tissue extracts for immunoblot analysis

50 to 100 mg of powdered frozen skeletal muscle or brain from 4 or 10-month-old mice was homogenized for 25 to 30 s with a Tissue Tearer in 15 volumes of homogenization buffer (HB) containing 50 mM Tris-HCl, pH 7.8, 10 mM EDTA, 2 mM EGTA, 100 mM NaF, 10 μg/ml leupeptin, 1 mM benzamidine, 0.1 mM Nα-p-tosyl-l-lysine chloromethyl ketone (TLCK), 0.5 mM phenylmethylsulfonyl fluoride (PMSF), 1 mM sodium orthovanadate, 0.4% (v/v) β-mercaptoethanol and 0.2% (v/v) Triton X-100. The homogenates were centrifuged at 6000*g* for 10 min to obtain a low-speed supernatant (LSS) and a low-speed pellet (LSP) as previously describe (28, 48). The LSP fractions were resuspended in the same volume of homogenization buffer as the LSS. For immunoblotting 30 to 40 μg of LSS protein and equivalent volumes of LSP were analyzed. Samples were separated on 10% SDS-PAGE gels at 200 V and transferred onto a nitrocellulose membrane at 100 V for 1.5 h or at 20 V for 20 h at 4 °C. The membranes were stained with Ponceau Red, destained, and blocked using Tris-buffered saline with 0.1% Tween 20 detergent (TBST) and 5% non-fat dry milk for 2 h at room temperature. The membranes were probed with antibodies against c-myc, Laforin, GYS, or PYGM at 4 °C for 20 h followed by incubation with anti-mouse/rabbit horseradish peroxidase-conjugated secondary antibody (Sigma) for 1 h at room temperature. The binding of the antibody was detected by enhanced chemiluminescence (Pierce ECL Western Blotting Substrate, Thermo Scientific). Autoradiograms were scanned (Epson Perfection V700 Photo) and band intensity was analyzed by using the digitized imaging analysis software UN-SCAN-IT gel (Silk Scientific).

### Immunoprecipitation

For immunoprecipitation, 200 to 400 mg of powdered frozen mouse skeletal muscle or brain from 4-month-old animals were homogenized on ice with a tissue Tearer in 5 or 10 volumes of homogenization buffer (HB) with 0.5% Triton X-100 without β-mercaptoethanol and the homogenates were centrifuged at 6000*g* for 10 min. The supernatants were pre-cleared by incubation with Protein-G Agarose (60 μl of a 50% slurry) for 1 h followed by centrifugation at 6000*g* for 10 min. The resulting supernatants were then incubated with c-myc antibodies or c-myc antibodies conjugated to Agarose beads (50 μl of a 50% slurry) and placed for 20 h at 4 °C on a Nutator mixer. The agarose beads were recovered by centrifugation at 25*g* for 30 s followed by three washes with HB and three washes with 25 mM Tris HCl, pH 7.4. A portion of the beads (20%, approximately 10 μl), was eluted with 1× SDS loading buffer for immunoblot analyses together with 30 μg protein of the Input (LSS) and the protein fraction unbound to the agarose beads. The amount of immunoprecipitated protein loaded could not be quantitated and was not detectable by Ponceau S staining of the membrane. The different amounts of protein loaded likely affect the apparent change in electrophoretic mobility of laforin and GYS1. The remainder of the agarose beads was processed for mass spectrometry. All immunoprecipitations were carried on the LSS of tissues from 4-month-old mice when most of the glycogen and glycogen metabolizing enzymes were in the LSS fraction (28, 48).

### Mass spectrometry

Sample preparation, mass spectrometry analysis, bioinformatics, and partial data evaluation for quantitative proteomics were performed at the Indiana University School of Medicine Center for Proteome Analysis following published protocols (96, 97, 98, 99).

### Sample preparation

Samples of c-myc immunoprecipitates from three biological replicates for each of the various genotypes, WT, malin-myc, malin-myc/LKO, LKO, malin-myc/LCS and LCS, were subjected to mass spectrometry analyses. Following immunoprecipitation and washing, the anti-c-myc agarose beads were further washed three times with 25 mM Tris HCl, pH 7.4, and then the anti-c-myc agarose beads were treated with 8 M Urea, 100 mM Tris-HCl, pH 8.5, and 5 mM Tris-(2-carboxyethyl)-phosphine hydrochloride (TCEP) for 30 min at room temperature to reduce the disulfide bonds. The resulting free cysteine thiols were alkylated using 10 mM chloroacetamide (CAA, Sigma Aldrich Cat No: C0267) for 30 min at RT, while protected from light. Samples were diluted to 2 M Urea with 50 mM Tris-HCl, pH 8.5 and proteolytic digestion was carried out overnight at 35 °C with Trypsin/LysC Gold (0.3 μg) Mass Spectrometry grade, Promega Corporation Cat No: V5072). Reactions were quenched with 0.5% (v/v) formic acid (FA) and centrifuged at 14,000*g* for 10 min prior to LC-MS/MS analysis.

### LC-MS/MS procedure

Samples were analyzed using a 5 cm trap column and 15 cm (2 μm particle size, 50 μm diameter) EasySpray (Thermo Fisher Scientific, 801A) analytical column on an UltiMate 3000 HPLC and Q-Exactive Plus mass spectrometer (Thermo Fisher Scientific). After loading the trap column for 5 min, the analytical column was switched online and solvent B was increased from 5% to 28% over 75 min, to 35% over 5 min, to 65% over 5 min and back to 5% over 5 min (Solvent A: 95% water, 5% acetonitrile, 0.1% formic acid; Solvent B: 100% acetonitrile, 0.1% formic acid). A data-dependent top 15 method acquisition method was used with a minimum AGC of 8 × 10^3^, charge exclusion of 1, and ≥7, and a dynamic exclusion of 30 s. MS scan range was at 200 to 2000 m/z, resolution of 70,000, AGC target 3 × 10^6^, and maximum injection time (IT) of 100 ms. For MS2 settings were, fixed first mass of 100 m/z, normalized collision energy of 30, isolation window of 2 m/z, resolution of 17,500, target AGC of 1 × 10^5^, and maximum IT of 50 ms.

### Proteomics data analysis

The MS raw data were analyzed by Proteome Discoverer 2.5 (Thermo Fisher Scientific) using SEQUEST HT. Data were searched against a *Mus musculus* protein database of 49,922 entries downloaded from Uniprot (01/09/2017), which included 73 frequently observed contaminants such as human keratins, immunoglobulins, and proteolytic enzymes. The parameters used for the SEQUEST HT search were the following: (full) trypsin digest with a maximum of three missed cleavages, precursor mass tolerance set to 10 ppm, and the fragment mass tolerance set to 0.02 Da. Dynamic modifications included methionine oxidation; phosphorylation on serine, threonine, and tyrosine residues, and in some searches either acetylation of lysine or ubiquitin remnant (+114.043 Da) on lysine; Protein N-terminal Met-loss, Acetylation, and Met-loss plus acetylation. Carbamidomethylation of cysteine was included as a static modification. The Percolator false discovery rate (FDR) was set to ≤1% for both the peptide-spectrum match and protein levels and the identification probability was >95% as previously described (97). The resulting peptide spectral matches were loaded into Scaffold 4 (Proteome Software) for visualization and further filtering. Proteins were only considered if they had a minimum of two unique peptides, and a peptide and protein threshold of 95 and 99%, respectively. The information about the proteins identified by LC-MS/MS in all c-myc and control immunoprecipitations for all the genotypes analyzed is provided in Supplementary Files 1 and 3–6.

### Protein analysis and quantitation

The total spectral counts (TSCs) that passed the above filtering criteria were used to calculate the Normalized Spectrum Abundance Factor (NSAF) of the proteins identified in each of the immunoprecipitations as previously described (97, 100, 101, 102). The NSAF values were calculated after the removal of the contaminant proteins described above (Supplementary Files 1–6). For quantitative analyses of proteins enriched in the c-myc immunoprecipitates, proteins were considered nonspecific and removed if the NSAF was higher in the controls, that did not have c-myc-tagged malin; if they were identified only in one of the three replicas and, where possible, if they were not tissue specific. Only proteins that were identified with an FC >1 (fold change) as compared to controls were included in the evaluation of statistical significance and enrichment of protein identified in the c-myc immunoprecipitates and used in DAVID Gene Ontology searches (Supplementary Files 1 and Enrichment File 2).

### Database for Annotation, Visualization and Integrated Discovery (DAVID Gene Ontology) and Volcano plot analyses

Gene Ontology functional enrichment analysis, using the DAVID web server (78, 79) was performed to evaluate the over-representation of biologically relevant groups of proteins and the pathways in which the identified proteins are involved. Gene lists were selected based on a *p*-value threshold of *p* ≤ 0.05 and a 2-fold enrichment threshold in the c-myc pull-downs of malin-myc and WT and entered into the DAVID interface. When the spectral counts were 0, a value of 0.001 was added for FC calculation. Default settings were used to identify clusters of gene products that are significantly enriched within known functional annotations (Fig. 3*A*; Supplementary Files 1 and Enrichment File 2). Volcano Plots (103) were constructed using the ggplot2package (104) operated within the R-studio suite (v4.3.2) to illustrate the statistically significant abundance of the proteins identified in the c-myc pulldown *versus* control. The plots utilize the Log_2_ of the fold-change (log_2_(FC) of the NSAF on the x-axis (c-myc pulled down proteins *versus* control or comparative samples) *versus* −log_10_ of the *p*-value (−log_10_(*p*-value), *t* test comparing c-myc pulled down proteins *versus* control or comparative samples) on the y-axis. Threshold values for the fold change (log_2_(FC) >1) and the significance (log_10_(*p*-value) >1.3) are indicated by dotted lines. Data in the upper right quadrants indicate enriched, those in the upper left, decreased and those in the middle, unchanged. Glycogen metabolizing proteins are annotated.

### Glycogen assay

Glycogen was measured as previously described (20, 48, 105). In brief, frozen tissues were hydrolyzed with 30% KOH in a boiling water bath for 30 min. After cooling, glycogen was precipitated in 66% ethanol with 15 mM LiCl and 2% Na_2_SO_4_. After three sequential precipitation, the pellets were dissolved in 0.2 M sodium acetate, pH 4.8 containing 0.3 mg/ml amyloglucosidase, and incubated for 20 h at 40 °C. Released glucose was converted to glucose-6-phosphate with hexokinase, oxidized in the presence of glucose-6-phosphate dehydrogenase/NADP^+,^ and the resulting absorbance was measured at 340 nm by the method of Bergmeyer (106). Glycogen content is quantitated against a glycogen standard curve and is expressed as μmol glucose per gram of wet tissue.

### Other methods

Glycogen phosphate and chain length were measured as previously described (28, 75, 107). Periodic acid-Schiff diastase-resistant (PASD) staining of muscle, heart, and brain sections was performed as previously described (48). Protein was measured by the Bradford method (108) using Bio-Rad dye reagent and bovine serum albumin as standard.

#### Statistics

Data are presented as mean ± SEM. Statistical significance was evaluated by using unpaired Student *t* test and considered significant at *p* < 0.05. One asterisk ∗*p* < 0.05, two asterisks ∗∗*p* < 0.01 *versus* WT or malin-myc.

## Data availability

All data are included in the manuscript or the supporting information. Mouse models will be available upon request.

## Supporting information

This article contains supporting information.

## Conflict of interest

The authors declare the following financial interests/personal relationships which may be considered as potential competing interests: T. D. H., A. A. D.-R., and P. J. R. were consultants for Maze Therapeutics. T. D. H. is also a consultant for SAJE Pharma. The other authors declare that they have no competing interest. None of the work in this article was supported by these entities.
